# Supplementary material for: Using Multiplex Amplicon PCR Technology to Efficiently and Timely Generate Rift Valley Fever Virus Sequence Data for Genomic Surveillance
Source: Viruses. 2023 Feb 9;15(2):477. doi: 10.3390/v15020477 (PMC9961268; doi:10.3390/v15020477)
Supplement: Supplementary file 1 [file viruses-15-00477-s001.zip › viruses-2188366-Supplementary_materials.pdf]

Supplementary materials

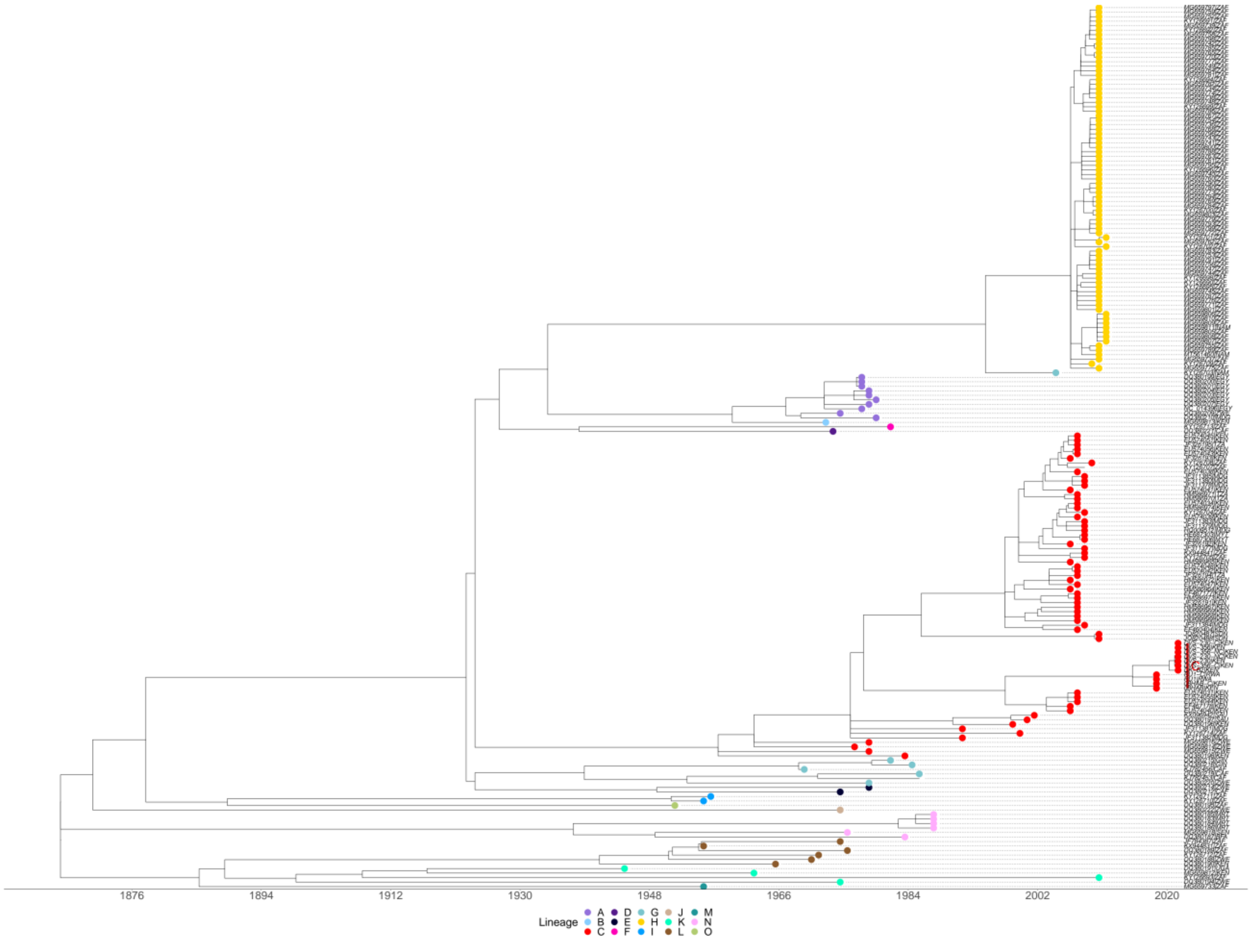


**Figure S1**. **Molecular clock phylogenetic reconstruction.** Time scaled phylogenetic tree showing the occurrence of lineages from 1951 to 2021. The tips of the phylogenetic tree are colored according to the lineages and are labeled as accession, country code and year.

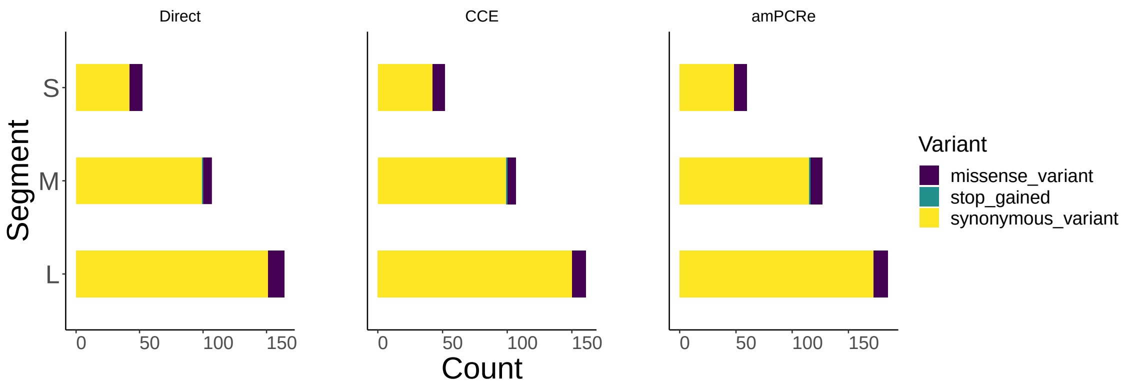


| | Chrom | Pos | Ref | Alt | Variant | SNP | Gene | AA.change | Segment |
|---|---|---|---|---|---|---|---|---|---|
| 1 | NC_014397.1 | 764 | G | A | missense_variant | G764A | RdRp | Arg256Lys | L |
| 2 | NC_014397.1 | 851 | G | A | missense_variant | G851A | RdRp | Ser285Asn | L |
| 3 | NC_014397.1 | 922 | G | A | missense_variant | G922A | RdRp | Val308Ile | L |
| 4 | NC_014397.1 | 1063 | G | A | missense_variant | G1063A | RdRp | Val355Met | L |
| 5 | NC_014397.1 | 1250 | G | A | missense_variant | G1250A | RdRp | Ser418Asn | L |
| 6 | NC_014397.1 | 1354 | G | A | missense_variant | G1354A | RdRp | As452Asn | L |
| 7 | NC_014397.1 | 2005 | G | A | missense_variant | G2005A | RdRp | Ala669Thr | L |
| 8 | NC_014397.1 | 5335 | A | G | missense_variant | A5335G | RdRp | Ile1779Val | L |
| 9 | NC_014397.1 | 5735 | A | G | missense_variant | A5735G | RdRp | Lys1913Arg | L |
| 10 | NC_014397.1 | 5968 | G | A | missense_variant | G5968A | RdRp | As1990Asn | L |
| 11 | NC_014396.1 | 715 | T | A | missense_variant | T715A | Gn | Leu239Gln | M |
| 12 | NC_014396.1 | 1717 | A | G | missense_variant | A1717G | Gn | As573Gly | M |
| 13 | NC_014396.1 | 1911 | A | G | missense_variant | A1911G | Gn | Ile638Val | M |
| 14 | NC_014396.1 | 1913 | A | G | missense_variant | A1913G | Gn | Ile639Met | M |
| 15 | NC_014396.1 | 3568 | C | T | missense_variant | C3568T | Gc | Thr1190Ile | M |
| 16 | NC_014395.1 | 101 | T | A | missense_variant | T101A | NSs | Phe35Ile | S |
| 17 | NC_014395.1 | 105 | G | A | missense_variant | G105A | NSs | Arg36Lys | S |
| 18 | NC_014395.1 | 432 | A | G | missense_variant | A432G | NSs | Asn145Ser | S |
| 19 | NC_014395.1 | 534 | C | T | missense_variant | C534T | NSs | Ala179Val | S |
| 20 | NC_014395.1 | 651 | C | T | missense_variant | C651T | NSs | Ser218Phe | S |
| 21 | NC_014395.1 | 684 | T | C | missense_variant | T684C | NSs | Val229Ala | S |
| 22 | NC_014395.1 | 758 | A | G | missense_variant | A758G | NSs | Ile254Val | S |
| 23 | NC_014395.1 | 764 | C | T | missense_variant | C764T | NSs | Pro256Ser | S |
| 24 | NC_014395.1 | 792 | A | G | missense_variant | A792G | NSs | Glu265Gly | S |
| 25 | NC_014395.1 | 1177 | C | T | missense_variant | C1177T | NP | Gly393Glu | S |

**Figure S2**. **SNPs annotation and summary.** The upper panel shows bar graphs of the SNPs observed in Direct, CCE and amPCRe treated samples. The graphs show the number of SNPs per segment as well as their annotations as synonymous or non-synonymous (stop-gained and missense). The lower panel is a summarized table showing the shared/common SNPs identified in all the three treatments for all the genomic segments.

**Table S1. Sample metadata sheet containing sequencing metrics, geographic information, and ELISA results for outbreak samples.**

| *Sample* | *Ct*[1] | *Reads* | *%RVFV*[2] | *Coverage* | *Depth*[3] | *Treatment* | *Segment* | *Host* | *Country* | *OD1* | *OD2* |
|---|---|---|---|---|---|---|---|---|---|---|---|
| *DK-B2* | 30.6 | 2940000 | 5.1 | 92 | 2105 | amPCRe | L | Cow | Kenya | 0.067 | 0.29 |
| *DK-B2* | 30.6 | 886000 | 0.1 | 24.2 | 6 | Direct | L | Cow | Kenya | 0.067 | 0.29 |
| *DVS-230* | 25.2 | 3960000 | 13.9 | 95.5 | 6032 | amPCRe | L | Cow | Kenya | 0.121 | 1.228 |
| *DVS-230* | 25.2 | 1668000 | 29.9 | 99.9 | 16019 | CCE | L | Cow | Kenya | 0.121 | 1.228 |
| *DVS-230* | 25.2 | 2848000 | 14.2 | 99.9 | 6811 | Direct | L | Cow | Kenya | 0.121 | 1.228 |
| *DVS-356* | 22.7 | 3640000 | 48.4 | 95.4 | 24230 | amPCRe | L | Cow | Kenya | 0.079 | 1.151 |
| *DVS-356* | 22.7 | 832000 | 21.4 | 99.7 | 5618 | CCE | L | Cow | Kenya | 0.079 | 1.151 |
| *DVS-356* | 22.7 | 1118000 | 41.9 | 99.9 | 8061 | Direct | L | Cow | Kenya | 0.079 | 1.151 |
| *DK-B2* | 30.6 | 2940000 | 87.1 | 91.6 | 61286 | amPCRe | M | Cow | Kenya | 0.067 | 0.29 |
| *DK-B2* | 30.6 | 886000 | 0 | 32.2 | 8 | Direct | M | Cow | Kenya | 0.067 | 0.29 |
| *DVS-230* | 25.2 | 3960000 | 25.7 | 95.4 | 18996 | amPCRe | M | Cow | Kenya | 0.121 | 1.228 |
| *DVS-230* | 25.2 | 1668000 | 10 | 99.6 | 8825 | CCE | M | Cow | Kenya | 0.121 | 1.228 |
| *DVS-230* | 25.2 | 2848000 | 6 | 99.8 | 4738 | Direct | M | Cow | Kenya | 0.121 | 1.228 |
| *DVS-356* | 22.7 | 3640000 | 34.4 | 98.8 | 28541 | amPCRe | M | Cow | Kenya | 0.079 | 1.228 |
| *DVS-356* | 22.7 | 832000 | 6.2 | 99.6 | 2651 | CCE | M | Cow | Kenya | 0.079 | 1.151 |
| *DVS-356* | 22.7 | 1118000 | 18 | 99.7 | 5608 | Direct | M | Cow | Kenya | 0.079 | 1.151 |
| *DK-B2* | 30.6 | 2940000 | 5.8 | 93.4 | 9172 | amPCRe | S | Cow | Kenya | 0.067 | 0.29 |
| *DK-B2* | 30.6 | 886000 | 0 | 72.7 | 18 | Direct | S | Cow | Kenya | 0.067 | 0.29 |
| *DVS-230* | 25.2 | 3960000 | 8.6 | 94.6 | 14117 | amPCRe | S | Cow | Kenya | 0.121 | 1.228 |
| *DVS-230* | 25.2 | 1668000 | 24 | 99.8 | 48512 | CCE | S | Cow | Kenya | 0.121 | 1.228 |
| *DVS-230* | 25.2 | 2848000 | 42.5 | 100 | 78662 | Direct | S | Cow | Kenya | 0.121 | 1.228 |
| *DVS-356* | 22.7 | 3640000 | 4 | 97 | 7306 | amPCRe | S | Cow | Kenya | 0.079 | 1.151 |
| *DVS-356* | 22.7 | 832000 | 15 | 99.4 | 14815 | CCE | S | Cow | Kenya | 0.079 | 1.151 |
| *DVS-356* | 22.7 | 1118000 | 22.4 | 99.9 | 16335 | Direct | S | Cow | Kenya | 0.079 | 1.151 |

[1]qRT-PCR cycle threshold values

[2]Percentage number of reads aligning to the Rift Valley fever reference genome segment

[3]Mean genome depth of coverage per genomic segment

**Table S2. Multiplex PCR amplicon primer scheme for the L-segment.** The first column is the reference sequence accession, second column is the start site, third column is the end site, fourth column is the primer name (depicted as either forward, LEFT suffix or reverse depicted with the RIGHT suffix), the fifth column is the pool and the sixth column is the strand.

| | | | | | |
|---|---|---|---|---|---|
| NC_014397.1 | 19 | 47 | rvfv-L-400_1_LEFT | 1 | + |
| NC_014397.1 | 394 | 416 | rvfv-L-400_1_RIGHT | 1 | - |
| NC_014397.1 | 332 | 355 | rvfv-L-400_2_LEFT | 2 | + |
| NC_014397.1 | 704 | 731 | rvfv-L-400_2_RIGHT | 2 | - |
| NC_014397.1 | 642 | 664 | rvfv-L-400_3_LEFT | 1 | + |
| NC_014397.1 | 1022 | 1044 | rvfv-L-400_3_RIGHT | 1 | - |
| NC_014397.1 | 948 | 970 | rvfv-L-400_4_LEFT | 2 | + |
| NC_014397.1 | 1339 | 1361 | rvfv-L-400_4_RIGHT | 2 | - |
| NC_014397.1 | 1256 | 1280 | rvfv-L-400_5_LEFT | 1 | + |
| NC_014397.1 | 1636 | 1660 | rvfv-L-400_5_RIGHT | 1 | - |
| NC_014397.1 | 1564 | 1587 | rvfv-L-400_6_LEFT | 2 | + |
| NC_014397.1 | 1932 | 1954 | rvfv-L-400_6_RIGHT | 2 | - |
| NC_014397.1 | 1861 | 1883 | rvfv-L-400_7_LEFT | 1 | + |
| NC_014397.1 | 2239 | 2259 | rvfv-L-400_7_RIGHT | 1 | - |
| NC_014397.1 | 2164 | 2186 | rvfv-L-400_8_LEFT | 2 | + |
| NC_014397.1 | 2545 | 2568 | rvfv-L-400_8_RIGHT | 2 | - |
| NC_014397.1 | 2480 | 2504 | rvfv-L-400_9_LEFT | 1 | + |

| | | | | | |
|---|---|---|---|---|---|
| NC_014397.1 | 2853 | 2876 | rvfv-L-400_9_RIGHT | 1 | - |
| NC_014397.1 | 2778 | 2800 | rvfv-L-400_10_LEFT | 2 | + |
| NC_014397.1 | 3168 | 3193 | rvfv-L-400_10_RIGHT | 2 | - |
| NC_014397.1 | 3094 | 3120 | rvfv-L-400_11_LEFT | 1 | + |
| NC_014397.1 | 3474 | 3495 | rvfv-L-400_11_RIGHT | 1 | - |
| NC_014397.1 | 3403 | 3427 | rvfv-L-400_12_LEFT | 2 | + |
| NC_014397.1 | 3790 | 3812 | rvfv-L-400_12_RIGHT | 2 | - |
| NC_014397.1 | 3724 | 3746 | rvfv-L-400_13_LEFT | 1 | + |
| NC_014397.1 | 4110 | 4133 | rvfv-L-400_13_RIGHT | 1 | - |
| NC_014397.1 | 4047 | 4069 | rvfv-L-400_14_LEFT | 2 | + |
| NC_014397.1 | 4423 | 4452 | rvfv-L-400_14_RIGHT | 2 | - |
| NC_014397.1 | 4363 | 4387 | rvfv-L-400_15_LEFT | 1 | + |
| NC_014397.1 | 4731 | 4758 | rvfv-L-400_15_RIGHT | 1 | - |
| NC_014397.1 | 4657 | 4681 | rvfv-L-400_16_LEFT | 2 | + |
| NC_014397.1 | 5041 | 5067 | rvfv-L-400_16_RIGHT | 2 | - |
| NC_014397.1 | 4968 | 4993 | rvfv-L-400_17_LEFT | 1 | + |
| NC_014397.1 | 5355 | 5377 | rvfv-L-400_17_RIGHT | 1 | - |
| NC_014397.1 | 5296 | 5319 | rvfv-L-400_18_LEFT | 2 | + |
| NC_014397.1 | 5674 | 5697 | rvfv-L-400_18_RIGHT | 2 | - |
| NC_014397.1 | 5602 | 5626 | rvfv-L-400_19_LEFT | 1 | + |
| NC_014397.1 | 5984 | 6008 | rvfv-L-400_19_RIGHT | 1 | - |
| NC_014397.1 | 5906 | 5935 | rvfv-L-400_20_LEFT | 2 | + |
| NC_014397.1 | 6279 | 6302 | rvfv-L-400_20_RIGHT | 2 | - |

**Table S3. Multiplex PCR amplicon primer scheme for the M-segment.** The first column is the reference sequence accession, second column is the start site, third column is the end site, fourth column is the primer name (depicted as either forward, LEFT suffix or reverse depicted with the RIGHT suffix), the fifth column is the pool and the sixth column is the strand.

| | | | | | |
|---|---|---|---|---|---|
| NC_014396.1 | 0 | 24 | rvfv-M-400_1_LEFT | 1 | + |
| NC_014396.1 | 396 | 418 | rvfv-M-400_1_RIGHT | 1 | - |
| NC_014396.1 | 329 | 351 | rvfv-M-400_2_LEFT | 2 | + |
| NC_014396.1 | 708 | 730 | rvfv-M-400_2_RIGHT | 2 | - |
| NC_014396.1 | 638 | 664 | rvfv-M-400_3_LEFT | 1 | + |
| NC_014396.1 | 1025 | 1046 | rvfv-M-400_3_RIGHT | 1 | - |
| NC_014396.1 | 965 | 987 | rvfv-M-400_4_LEFT | 2 | + |
| NC_014396.1 | 1345 | 1368 | rvfv-M-400_4_RIGHT | 2 | - |
| NC_014396.1 | 1279 | 1301 | rvfv-M-400_5_LEFT | 1 | + |
| NC_014396.1 | 1667 | 1690 | rvfv-M-400_5_RIGHT | 1 | - |
| NC_014396.1 | 1581 | 1608 | rvfv-M-400_6_LEFT | 2 | + |

| | | | | | |
|---|---|---|---|---|---|
| NC_014396.1 | 1969 | 1991 | rvfv-M-400_6_RIGHT | 2 | - |
| NC_014396.1 | 1908 | 1932 | rvfv-M-400_7_LEFT | 1 | + |
| NC_014396.1 | 2292 | 2312 | rvfv-M-400_7_RIGHT | 1 | - |
| NC_014396.1 | 2217 | 2239 | rvfv-M-400_8_LEFT | 2 | + |
| NC_014396.1 | 2612 | 2636 | rvfv-M-400_8_RIGHT | 2 | - |
| NC_014396.1 | 2537 | 2560 | rvfv-M-400_9_LEFT | 1 | + |
| NC_014396.1 | 2934 | 2957 | rvfv-M-400_9_RIGHT | 1 | - |
| NC_014396.1 | 2867 | 2892 | rvfv-M-400_10_LEFT | 2 | + |
| NC_014396.1 | 3242 | 3268 | rvfv-M-400_10_RIGHT | 2 | - |
| NC_014396.1 | 3164 | 3186 | rvfv-M-400_11_LEFT | 1 | + |
| NC_014396.1 | 3551 | 3576 | rvfv-M-400_11_RIGHT | 1 | - |
| NC_014396.1 | 3471 | 3493 | rvfv-M-400_12_LEFT | 2 | + |
| NC_014396.1 | 3864 | 3885 | rvfv-M-400_12_RIGHT | 2 | - |

**Table S4. Multiplex PCR amplicon primer scheme for the S-segment.** The first column is the reference sequence accession, second column is the start site, third column is the end site, fourth column is the primer name (depicted as either forward, LEFT suffix or reverse depicted with the RIGHT suffix), the fifth column is the pool and the sixth column is the strand.

| | | | | | |
|---|---|---|---|---|---|
| NC_014395.1 | 0 | 22 | rvfv-S-400_1_LEFT | 1 | + |
| NC_014395.1 | 386 | 408 | rvfv-S-400_1_RIGHT | 1 | - |
| NC_014395.1 | 328 | 350 | rvfv-S-400_2_LEFT | 2 | + |
| NC_014395.1 | 709 | 731 | rvfv-S-400_2_RIGHT | 2 | - |
| NC_014395.1 | 641 | 667 | rvfv-S-400_3_LEFT | 1 | + |
| NC_014395.1 | 1031 | 1054 | rvfv-S-400_3_RIGHT | 1 | - |
| NC_014395.1 | 957 | 979 | rvfv-S-400_4_LEFT | 2 | + |
| NC_014395.1 | 1339 | 1361 | rvfv-S-400_4_RIGHT | 2 | - |
| NC_014395.1 | 1265 | 1286 | rvfv-S-400_5_LEFT | 1 | + |
| NC_014395.1 | 1658 | 1684 | rvfv-S-400_5_RIGHT | 1 | - |

**Table S5. Multiplex PCR amplicon primers for sequencing RVFV.** S (*n*=10), M (*n*=24), L (*n*=40). The odd numbered primers belong to pool 1 while even numbered primers belong to pool 2. The primers were designed to amplify a target sequence of approximately 400 bases within the RVFV genome. Reference genomes are used to select primer pairs that maximize the likelihood of successful amplification for known virus diversity. A pairwise local alignment score between each candidate and the reference is calculated to ensure the most 'universal' candidate primers are picked for the scheme.

| *Primer* | *name* | *pool* | *seq* | *%gc* | *Melting temperature (use 65)* |
|---|---|---|---|---|---|
| *rvfv-S-400_1_LEFT* | 1 | ACACAAAGACCCCCTAGTGCTT | 22 | 50.00 | 61.75 |
| *rvfv-S-400_1_RIGHT* | 1 | AGGTCAAAGAAAGCCAGTGAGG | 22 | 50.00 | 60.67 |
| *rvfv-S-400_2_LEFT* | 2 | AGTGATTTCACAAGGTTCCCCA | 22 | 45.45 | 60.01 |
| *rvfv-S-400_2_RIGHT* | 2 | TCATGCTAGGAAGTGATGAGCG | 22 | 50.00 | 60.40 |
| *rvfv-S-400_3_LEFT* | 1 | AGGACATTTCTAATGCTGTAGTTCCA | 26 | 38.46 | 60.74 |
| *rvfv-S-400_3_RIGHT* | 1 | TGAATGCAGCAGTGAATAGCAAC | 23 | 43.48 | 60.37 |
| *rvfv-S-400_4_LEFT* | 2 | GCTGACGGCTTCCCATTAGAAT | 22 | 50.00 | 60.93 |
| *rvfv-S-400_4_RIGHT* | 2 | TCCCGGGATGAGTTGACTCTAT | 22 | 50.00 | 60.28 |
| *rvfv-S-400_5_LEFT* | 1 | AGTGACAGGAAGCCACTCACT | 21 | 52.38 | 61.14 |
| *rvfv-S-400_5_RIGHT* | 1 | AGCTCCCTAGAGATACAAACACTATT | 26 | 38.46 | 59.61 |
| *rvfv-M-400_1_LEFT* | 1 | ACACAAAGACGGTGCATTAAATGT | 24 | 37.50 | 60.28 |
| *rvfv-M-400_1_RIGHT* | 1 | CCTGCCATGGTTTCTCTCCCTA | 22 | 54.55 | 61.68 |
| *rvfv-M-400_2_LEFT* | 2 | AAGGGTCCTCCTCACAAAAAGC | 22 | 50.00 | 60.94 |
| *rvfv-M-400_2_RIGHT* | 2 | TTCCCATGAGCACTCTGAAGGT | 22 | 50.00 | 61.55 |
| *rvfv-M-400_3_LEFT* | 1 | GCTCATCATAGAACTCTACTAGAGGC | 26 | 46.15 | 60.56 |
| *rvfv-M-400_3_RIGHT* | 1 | AACCTCATGTGCAGTGCTGAG | 21 | 52.38 | 60.98 |
| *rvfv-M-400_4_LEFT* | 2 | ACAAAAATGAAAGGGGTCTGCG | 22 | 45.45 | 60.14 |
| *rvfv-M-400_4_RIGHT* | 2 | CCCCTGATACTTGTATCTGCACA | 23 | 47.83 | 60.12 |
| *rvfv-M-400_5_LEFT* | 1 | CTCTGCTTATGAGTGCACTGCT | 22 | 50.00 | 60.86 |
| *rvfv-M-400_5_RIGHT* | 1 | GGGCAGTGAGCTACTATTTTGGA | 23 | 47.83 | 60.63 |
| *rvfv-M-400_6_LEFT* | 2 | AGATTACACTCAAGTATCCAGGGATAT | 27 | 37.04 | 59.55 |
| *rvfv-M-400_6_RIGHT* | 2 | CTGACCTCCTTCCATCCATCCT | 22 | 54.55 | 61.15 |
| *rvfv-M-400_7_LEFT* | 1 | GGGTGTATAAGAAGATGGTTGCCA | 24 | 45.83 | 60.95 |
| *rvfv-M-400_7_RIGHT* | 1 | AGTCCAATAGCTCTGGCCCT | 20 | 55.00 | 60.34 |
| *rvfv-M-400_8_LEFT* | 2 | TGTTGAAGGGGGGTCAAGGAAGA | 22 | 50.00 | 61.42 |
| *rvfv-M-400_8_RIGHT* | 2 | TGTTGAAACAGAGCCATCAAAGTC | 24 | 41.67 | 60.28 |
| *rvfv-M-400_9_LEFT* | 1 | CAGTCAGTTAGAAAAGAGGCCCT | 23 | 47.83 | 60.25 |

| | | | | | |
|---|---|---|---|---|---|
| *rvfv-M-400_9_RIGHT* | 1 | ACCCCTCTCAAAGACAACAAAGG | 23 | 47.83 | 60.95 |
| *rvfv-M-400_10_LEFT* | 2 | GCACCAAACCTTATCTCATACAAGC | 25 | 44.00 | 60.83 |
| *rvfv-M-400_10_RIGHT* | 2 | CACTGATCTTTTGTTCCATTCTCTGA | 26 | 38.46 | 60.01 |
| *rvfv-M-400_11_LEFT* | 1 | TGCCTGTCTATCACATCCACAG | 22 | 50.00 | 60.01 |
| *rvfv-M-400_11_RIGHT* | 1 | AGAGGCCTGTTCTTCCAAGATATAT | 25 | 40.00 | 59.54 |
| *rvfv-M-400_12_LEFT* | 2 | GTTGGTTTGGAGGGCCTCTTAA | 22 | 50.00 | 60.67 |
| *rvfv-M-400_12_RIGHT* | 2 | ACACAAAGACCGGTGCAACTT | 21 | 47.62 | 61.06 |
| *rvfv-L-400_1_LEFT* | 1 | TGGATTCTATATTATCAAAACAGCTGGT | 28 | 32.14 | 59.61 |
| *rvfv-L-400_1_RIGHT* | 1 | TCATCTCCCCTAAAGGTGGTGA | 22 | 50.00 | 60.41 |
| *rvfv-L-400_2_LEFT* | 2 | TGATCATTTGAGCCCTGACATGA | 23 | 43.48 | 60.31 |
| *rvfv-L-400_2_RIGHT* | 2 | ACAGATTCTGTGACTGACCAATTTATT | 27 | 33.33 | 59.83 |
| *rvfv-L-400_3_LEFT* | 1 | TCCACAGATGAGGAACTAGGGA | 22 | 50.00 | 59.87 |
| *rvfv-L-400_3_RIGHT* | 1 | GCACAGATCTTTGCCATCGACA | 22 | 50.00 | 61.69 |
| *rvfv-L-400_4_LEFT* | 2 | GCAGAAGACAACCTTAGGGACC | 22 | 54.55 | 60.80 |
| *rvfv-L-400_4_RIGHT* | 2 | TCCACATCATGATCTGGGGAGA | 22 | 50.00 | 60.55 |
| *rvfv-L-400_5_LEFT* | 1 | CATAGCTGCTTTAGGGGTTAATGG | 24 | 45.83 | 59.80 |
| *rvfv-L-400_5_RIGHT* | 1 | AAATCCCAGAACCTAGTAGTCGTT | 24 | 41.67 | 59.55 |
| *rvfv-L-400_6_LEFT* | 2 | TGGTCAGTTTGATAGGAGCTGAG | 23 | 47.83 | 60.06 |
| *rvfv-L-400_6_RIGHT* | 2 | CATCCATTGCTGCAGAGTCTGA | 22 | 50.00 | 60.86 |
| *rvfv-L-400_7_LEFT* | 1 | CCTCCTTCTCATTCTGGGCAGA | 22 | 54.55 | 61.67 |
| *rvfv-L-400_7_RIGHT* | 1 | AAGCAGTGGACGCCCTGAAA | 20 | 55.00 | 62.35 |
| *rvfv-L-400_8_LEFT* | 2 | AGAGAATAGCAGGTGAGCCCTT | 22 | 50.00 | 61.08 |
| *rvfv-L-400_8_RIGHT* | 2 | CCTTTCTAGAGTCAACCCACTGA | 23 | 47.83 | 59.68 |
| *rvfv-L-400_9_LEFT* | 1 | AGTCTTAAGGAGCCTCTATGGACA | 24 | 45.83 | 60.66 |
| *rvfv-L-400_9_RIGHT* | 1 | AAGAACTTCCCTATAGACCTGGC | 23 | 47.83 | 59.55 |
| *rvfv-L-400_10_LEFT* | 2 | CAGCATGGAGGTCTGAGAGAGA | 22 | 54.55 | 61.13 |
| *rvfv-L-400_10_RIGHT* | 2 | AGAGATCCATCACAAAGTCATCTCT | 25 | 40.00 | 59.73 |
| *rvfv-L-400_11_LEFT* | 1 | TGTTTACCAGGAAAAGGATGATGATG | 26 | 38.46 | 60.06 |
| *rvfv-L-400_11_RIGHT* | 1 | GCATATAGCTGCGGCCACTTT | 21 | 52.38 | 61.52 |
| *rvfv-L-400_12_LEFT* | 2 | TGCAAGGATCAGATGATAGTAGCA | 24 | 41.67 | 59.73 |
| *rvfv-L-400_12_RIGHT* | 2 | AATAGCTCAGACACTCCCATGC | 22 | 50.00 | 60.34 |
| *rvfv-L-400_13_LEFT* | 1 | GTGGGTCATTCTCCTTAGCTGC | 22 | 54.55 | 61.18 |
| *rvfv-L-400_13_RIGHT* | 1 | TCTGGTATGTTCAAACGGTCTCT | 23 | 43.48 | 59.68 |

| | | | | | |
|---|---|---|---|---|---|
| *rvfv-L-400_14_LEFT* | 2 | GGTGGCGCTCTAATTCTTAGCT | 22 | 50.00 | 60.66 |
| *rvfv-L-400_14_RIGHT* | 2 | CGGGAATAGGAATAATATATCTTCTGGCT | 29 | 37.93 | 61.03 |
| *rvfv-L-400_15_LEFT* | 1 | AGTACAGCTTGCTACAAAAGATGG | 24 | 41.67 | 59.62 |
| *rvfv-L-400_15_RIGHT* | 1 | CCTAACTTGAATGTGGTTATTCAGTGG | 27 | 40.74 | 60.85 |
| *rvfv-L-400_16_LEFT* | 2 | TGGCTGAATGGGAAAAGCTAAAGA | 24 | 41.67 | 61.08 |
| *rvfv-L-400_16_RIGHT* | 2 | GGTTTTTGTCTTACCATCTGATTCCT | 26 | 38.46 | 60.01 |
| *rvfv-L-400_17_LEFT* | 1 | GGCCCATATAGTGAGAGCTATAAGC | 25 | 48.00 | 60.83 |
| *rvfv-L-400_17_RIGHT* | 1 | TGTTTGTGACCCCCATGTCTTC | 22 | 50.00 | 60.93 |
| *rvfv-L-400_18_LEFT* | 2 | TCACAACTAACTGTCAGAGCAGC | 23 | 47.83 | 61.05 |
| *rvfv-L-400_18_RIGHT* | 2 | TGGCTCCCTCTCAAATAATTGCA | 23 | 43.48 | 60.57 |
| *rvfv-L-400_19_LEFT* | 1 | ACACTGCATCAGACAATGATCTCA | 24 | 41.67 | 60.65 |
| *rvfv-L-400_19_RIGHT* | 1 | TCCATCTCGTAAGGACCACTAACA | 24 | 45.83 | 61.14 |
| *rvfv-L-400_20_LEFT* | 2 | TGATGCGTTAATGGATCTAATGATAGAAG | 29 | 34.48 | 60.32 |
| *rvfv-L-400_20_RIGHT* | 2 | TGGTCTTAGCCTAGCATGTCATC | 23 | 47.83 | 60.18 |

**Table S6. Sequence accessions and metadata (n = 196) used in molecular phylogenetic analyses.** The Accession column is separated by '|' with the first string showing the accession from NCBI or strain of sequenced data in this study, the second part is the 3-letter country code and the third is the year of sample collection. The second column additionally provides the year of sample collection.

| *Accession* | *Country* |
|---|---|
| *DQ380187\|BFA\|1983* | BFA |
| *KJ782453\|CAF\|1985* | CAF |
| *DQ380212\|CAF\|1974* | CAF |
| *KJ782456\|CAF\|1969* | CAF |
| *DQ380219\|CAF\|1985* | CAF |
| *DQ380221\|CAF\|1973* | CAF |
| *DQ380205\|EGY\|1979* | EGY |
| *DQ380203\|EGY\|1978* | EGY |
| *DQ380201\|EGY\|1977* | EGY |
| *DQ380200\|EGY\|1977* | EGY |
| *DQ380199\|EGY\|1977* | EGY |
| *DQ380207\|EGY\|1978* | EGY |
| *DQ380204\|EGY\|1978* | EGY |
| *NC_014396\|EGY\|1977* | EGY |
| *DQ380216\|GIN\|1984* | GIN |
| *DQ380215\|GIN\|1981* | GIN |
| *EU574044\|KEN\|2007* | KEN |
| *EU574043\|KEN\|2007* | KEN |
| *EU574042\|KEN\|2007* | KEN |
| *EU574041\|KEN\|2006* | KEN |
| *EU574040\|KEN\|2006* | KEN |
| *EU574039\|KEN\|2007* | KEN |

| | |
|---|---|
| *EU574038\|KEN\|2007* | KEN |
| *EU574045\|KEN\|2007* | KEN |
| *EU574047\|KEN\|2007* | KEN |
| *08HAB\|KEN\|2018* | KEN |
| *EU574034\|KEN\|2007* | KEN |
| *HM586974\|KEN\|2007* | KEN |
| *HM586973\|KEN\|2007* | KEN |
| *HM586972\|KEN\|2006* | KEN |
| *JF326191\|KEN\|2007* | KEN |
| *JF326192\|KEN\|2006* | KEN |
| *HM586969\|KEN\|2007* | KEN |
| *EU574048\|KEN\|2007* | KEN |
| *HM586968\|KEN\|2007* | KEN |
| *HM586966\|KEN\|2007* | KEN |
| *HM586965\|KEN\|2006* | KEN |
| *HM586964\|KEN\|2006* | KEN |
| *EU574056\|KEN\|2007* | KEN |
| *EU574055\|KEN\|2007* | KEN |
| *EU574051\|KEN\|2007* | KEN |
| *HM586967\|KEN\|2007* | KEN |
| *EU574031\|KEN\|2007* | KEN |
| *EF460404\|KEN\|2007* | KEN |
| *EF467177\|KEN\|2007* | KEN |
| *08HAB-C\|KEN\|2018* | KEN |
| *DK-B2\|KEN\|2021* | KEN |
| *DQ380190\|KEN\|1965* | KEN |
| *EF467178\|KEN\|2006* | KEN |
| *MG659812\|KEN\|1962* | KEN |
| *DQ380196\|KEN\|1998* | KEN |
| *DQ380198\|KEN\|1983* | KEN |
| *MG659813\|KEN\|1972* | KEN |
| *DVS-230-NC\|KEN\|2021* | KEN |
| *JF326193\|KEN\|2006* | KEN |
| *DVS-356-C\|KEN\|2021* | KEN |
| *DVS-356\|KEN\|2021* | KEN |
| *DVS-356-NC\|KEN\|2021* | KEN |
| *DVS-230\|KEN\|2021* | KEN |
| *DVS-230-C\|KEN\|2021* | KEN |
| *JF311381\|MDG\|1991* | MDG |
| *JF311380\|MDG\|2008* | MDG |
| *JF311379\|MDG\|2008* | MDG |
| *JF311384\|MDG\|2008* | MDG |
| *JF311382\|MDG\|1991* | MDG |
| *JF311378\|MDG\|2008* | MDG |
| *JF311377\|MDG\|2008* | MDG |
| *JF311383\|MDG\|2008* | MDG |
| *HQ009512\|MDG\|2008* | MDG |
| *DQ380210\|MDG\|1979* | MDG |
| *JF311385\|MDG\|2008* | MDG |
| *DQ380184\|MRT\|1987* | MRT |
| *DQ380186\|MRT\|1987* | MRT |
| *DQ380183\|MRT\|1987* | MRT |
| *DQ380185\|MRT\|1987* | MRT |
| *HE687306\|MYT\|2008* | MYT |
| *HE687303\|MYT\|2008* | MYT |
| *MT561460\|NAM\|2010* | NAM |
| *MG659811\|NAM\|2011* | NAM |
| *KY126703\|NAM\|2004* | NAM |
| *RU1\|RWA\|2018* | RWA |

| | |
|---|---|
| *RU1-C\|RWA\|2018* | RWA |
| *DQ380197\|SAU\|2000* | SAU |
| *KX096942\|SAU\|2001* | SAU |
| *JQ820488\|SDN\|2010* | SDN |
| *JQ820487\|SDN\|2010* | SDN |
| *MG659818\|SEN\|1975* | SEN |
| *HM586970\|TZA\|2007* | TZA |
| *HM586971\|TZA\|2007* | TZA |
| *JF326195\|TZA\|2007* | TZA |
| *JF326194\|TZA\|2007* | TZA |
| *DQ380191\|UGA\|1944* | UGA |
| *MG659763\|ZAF\|2010* | ZAF |
| *MG659784\|ZAF\|2010* | ZAF |
| *MG659783\|ZAF\|2010* | ZAF |
| *MG659782\|ZAF\|2010* | ZAF |
| *MG659781\|ZAF\|2010* | ZAF |
| *MG659780\|ZAF\|2010* | ZAF |
| *MG659779\|ZAF\|2010* | ZAF |
| *MG659777\|ZAF\|2010* | ZAF |
| *MG659776\|ZAF\|2010* | ZAF |
| *MG659775\|ZAF\|2010* | ZAF |
| *MG659774\|ZAF\|2010* | ZAF |
| *MG659785\|ZAF\|2010* | ZAF |
| *MG659772\|ZAF\|2010* | ZAF |
| *MG659771\|ZAF\|2010* | ZAF |
| *MG659770\|ZAF\|2010* | ZAF |
| *MG659769\|ZAF\|2010* | ZAF |
| *MG659768\|ZAF\|2010* | ZAF |
| *MG659767\|ZAF\|2010* | ZAF |
| *MG659765\|ZAF\|2010* | ZAF |
| *MG659764\|ZAF\|2010* | ZAF |
| *MG659762\|ZAF\|2010* | ZAF |
| *MG659773\|ZAF\|2010* | ZAF |
| *MG659787\|ZAF\|2010* | ZAF |
| *MG659788\|ZAF\|2010* | ZAF |
| *DQ380195\|ZAF\|1951* | ZAF |
| *MG659810\|ZAF\|2011* | ZAF |
| *MG659809\|ZAF\|2011* | ZAF |
| *MG659808\|ZAF\|2011* | ZAF |
| *MG659807\|ZAF\|2011* | ZAF |
| *MG659806\|ZAF\|2011* | ZAF |
| *MG659805\|ZAF\|2011* | ZAF |
| *MG659803\|ZAF\|2010* | ZAF |
| *MG659801\|ZAF\|2010* | ZAF |
| *MG659800\|ZAF\|2010* | ZAF |
| *MG659799\|ZAF\|2010* | ZAF |
| *MG659798\|ZAF\|2010* | ZAF |
| *MG659797\|ZAF\|2010* | ZAF |
| *MG659796\|ZAF\|2010* | ZAF |
| *MG659795\|ZAF\|2010* | ZAF |
| *DQ380189\|ZAF\|1975* | ZAF |
| *MG659794\|ZAF\|2010* | ZAF |
| *MG659793\|ZAF\|2010* | ZAF |
| *MG659791\|ZAF\|2010* | ZAF |
| *MG659761\|ZAF\|2010* | ZAF |
| *MG659789\|ZAF\|2010* | ZAF |
| *MG659786\|ZAF\|2010* | ZAF |
| *MG659790\|ZAF\|2010* | ZAF |
| *MG659759\|ZAF\|2010* | ZAF |

| | |
|---|---|
| *MG659758\|ZAF\|2010* | ZAF |
| *KY126710\|ZAF\|1955* | ZAF |
| *KY126709\|ZAF\|2009* | ZAF |
| *MG659760\|ZAF\|2010* | ZAF |
| *KY126707\|ZAF\|2008* | ZAF |
| *KY126705\|ZAF\|2008* | ZAF |
| *KY126704\|ZAF\|2008* | ZAF |
| *KY126702\|ZAF\|2011* | ZAF |
| *KY126701\|ZAF\|2011* | ZAF |
| *KY126700\|ZAF\|2010* | ZAF |
| *KY126711\|ZAF\|1956* | ZAF |
| *KY126699\|ZAF\|2010* | ZAF |
| *KY126696\|ZAF\|2010* | ZAF |
| *KY126695\|ZAF\|2010* | ZAF |
| *KY126694\|ZAF\|2010* | ZAF |
| *KY126692\|ZAF\|2010* | ZAF |
| *KY126691\|ZAF\|2010* | ZAF |
| *KY126690\|ZAF\|2010* | ZAF |
| *KX944841\|ZAF\|2008* | ZAF |
| *KX944831\|ZAF\|1955* | ZAF |
| *JF784387\|ZAF\|1974* | ZAF |
| *KY126698\|ZAF\|2010* | ZAF |
| *KY126712\|ZAF\|1971* | ZAF |
| *KY126708\|ZAF\|2009* | ZAF |
| *KY126714\|ZAF\|1999* | ZAF |
| *MG659756\|ZAF\|2010* | ZAF |
| *MG659755\|ZAF\|2010* | ZAF |
| *MG659754\|ZAF\|2010* | ZAF |
| *MG659753\|ZAF\|2010* | ZAF |
| *MG659752\|ZAF\|2010* | ZAF |
| *MG659749\|ZAF\|2010* | ZAF |
| *KY126713\|ZAF\|1981* | ZAF |
| *MG659748\|ZAF\|2010* | ZAF |
| *MG659747\|ZAF\|2010* | ZAF |
| *MG659745\|ZAF\|2010* | ZAF |
| *KY126693\|ZAF\|2010* | ZAF |
| *MG659743\|ZAF\|2010* | ZAF |
| *MG659742\|ZAF\|2010* | ZAF |
| *MG659741\|ZAF\|2010* | ZAF |
| *MG659740\|ZAF\|2010* | ZAF |
| *MG659739\|ZAF\|2010* | ZAF |
| *MG659738\|ZAF\|2010* | ZAF |
| *MG659737\|ZAF\|2010* | ZAF |
| *MG659736\|ZAF\|2010* | ZAF |
| *MG659734\|ZAF\|2010* | ZAF |
| *MG659744\|ZAF\|2010* | ZAF |
| *MG659733\|ZAF\|1955* | ZAF |
| *DQ380188\|ZWE\|1970* | ZWE |
| *DQ380222\|ZWE\|1974* | ZWE |
| *MG659816\|ZWE\|1978* | ZWE |
| *DQ380214\|ZWE\|1978* | ZWE |
| *MG659814\|ZWE\|1976* | ZWE |
| *DQ380220\|ZWE\|1978* | ZWE |
| *DQ380194\|ZWE\|1974* | ZWE |
| *MG659815\|ZWE\|1978* | ZWE |
| *DQ380209\|ZWE\|1974* | ZWE |
